# Combination Effects of Plant Extracts Rich in Tannins and Saponins as Feed Additives for Mitigating in Vitro Ruminal Methane and Ammonia Formation

**DOI:** 10.3390/ani10091531

**Published:** 2020-08-30

**Authors:** Anuraga Jayanegara, Yogianto Yogianto, Elizabeth Wina, Asep Sudarman, Makoto Kondo, Taketo Obitsu, Michael Kreuzer

**Affiliations:** 1 Department of Animal Nutrition and Feed Technology, Faculty of Animal Science, IPB University, Bogor 16680, Indonesia; a_sudarman@yahoo.com; 2 Graduate School of Animal Nutrition and Feed Science, IPB University, Bogor 16680, Indonesia; myogianto@yahoo.com; 3 Indonesian Research Center for Animal Production, Ciawi Bogor 16002, Indonesia; winabudi@yahoo.com; 4 Department of Bioresources, Mie University, Tsu, Mie 514-8507, Japan; makok@bio.mie-u.ac.jp; 5 Graduate School of Integrated Sciences for Life, Hiroshima University, 1-4-4 Kagamiyama, Higashihiroshima 739-8528, Japan; tobitsu@hiroshima-u.ac.jp; 6 ETH Zurich, Institute of Agricultural Sciences, Universitätstrasse 2, 8092 Zurich, Switzerland; michael.kreuzer@usys.ethz.ch

**Keywords:** plant secondary compounds, methanogenesis, nitrogen, ruminants

## Abstract

**Simple Summary:**

Ruminant livestock contribute to global warming by emitting methane, a major greenhouse gas, as a product of microbial fermentation occurring in the rumen. Apart from its contribution to greenhouse gas emissions, methane emissions represent an energy loss in ruminants. Excessive ruminal ammonia formation, on the other hand, leads to a higher risk of pollution via ammonia, nitrous oxide, and nitrate emissions. Natural plant secondary compounds such as tannins, saponins, and essential oils are among the promising feed additives to mitigate enteric methane and ammonia formation. Though both tannins and saponins, when tested separately, have been reported to be effective, their combinations have rarely been tested. Therefore, in the present study, whether the combination of plant extracts rich in tannins and saponins would act additively or non-additively (associatively) in decreasing methane and ammonia formation in an artificial rumen system was investigated. Indeed, the addition of plant extracts rich in tannins and saponins, either individually or in combination, decreased the methane proportion of total gas in both high-forage and high-concentrate diets. This indicates their effectiveness as anti-methanogenic agents across contrasting diet types. Their effects were generally additive and occasionally synergistic (i.e., more than proportionate), especially in mitigating ruminal ammonia formation and, less clearly, concerning methane emissions.

**Abstract:**

The objective of this experiment was to test the effects of combining plant extracts rich in tannins and saponins at varying proportions on in vitro ruminal methane and ammonia formation. Tannins were extracted from *Swietenia mahogani* leaves and saponins from *Sapindus rarak* fruits with various solvents. The extracts obtained with the most efficient solvents (tannins: 75% water and 25% methanol; saponins: pure methanol) were then used in vitro. The treatments consisted of two substrate types (high-forage (HF) or high-concentrate (HC) diets) and five extract combinations (tannins: saponins, 1:0, 3:1, 1:1, 1:3, and 0:1) added at 2 mg/mL in incubation liquid. In vitro incubation was performed in four runs, with each treatment being represented with two replicates per run. The addition of plant extracts rich in tannins and saponins, either individually or in combination, decreased the methane proportion of total gas in both the HF (*p* < 0.05) and HC (*p* < 0.05) diets. The effects of the plant extracts rich in tannins and saponins were generally additive in mitigating methane emissions. Favorable associative effects between the extracts were observed in the ammonia concentration, both in the HF (*p* < 0.001) and HC (*p* < 0.01) diets and in the methane proportion of total gas, with a 1:3 mixture of tannins and saponins added to the HC diet (*p* < 0.05).

## 1. Introduction

The accumulation of various greenhouse gases, such as carbon dioxide, methane, and nitrous oxide, in the atmosphere has been considered to be a primary factor responsible for the current global warming phenomenon. Livestock, particularly ruminants, contribute to anthropogenic greenhouse gas emissions via methane as a product of microbial fermentation occurring in the rumen and, less so, in the hindgut and the manure [1,2]. Such enteric fermentation contributes to approximately 17% of global methane sources [3], and emission trends are increasing, particularly due to the contribution from developing regions [4]. Methane is formed by archaeal methanogens, predominantly from carbon dioxide and hydrogen as substrates using this mechanism to generate energy under anaerobic conditions [5]. Apart from its contribution to global warming, methane emissions represent an energy loss from livestock on the order of 6‒10% of the gross energy intake of ruminants, especially when forage-based diets are fed [6]. The Intergovernmental Panel on Climate Change (IPCC) [7] assumes a default loss of 6.5% of gross energy intake for such diets. Reducing methane may therefore improve the efficiency of energy utilization—but only when the mitigation accompanies unchanged energy digestibility. Another type of emission that is of great environmental concern is that of nitrogenous compounds, including ammonia, nitrate, and the greenhouse gas nitrous oxide. These compounds are not emitted directly by the animal; instead, they are formed in the manure from excessive urinary nitrogen. The latter is closely correlated with ammonia formation and absorption from the rumen [8]. Thus, any effective measures to mitigate methane and ammonia formation in the rumen of livestock would be beneficial.

A number of feed additives have been tested for their potential to mitigate methane emissions from ruminants [9,10]. Natural plant secondary compounds such as tannins, saponins, and essential oils are among the promising feed additives to mitigate enteric methane emission, and they have been repeatedly investigated [11,12,13,14,15]. For instance, extracted and purified tannins, either condensed (from mimosa and quebracho) or hydrolysable (from chestnut and sumac), added at levels of 0.5–1.0 mg/mL, were shown to substantially decrease the methane per unit of digestible organic matter and methanogen population [16]. Similarly, a methanolic extract of the *Saponaria officinalis* root (containing saponins) reduced methanogenesis by about 30% and lowered protozoa and methanogen counts without causing adverse effects on rumen fermentation and in vitro dry matter digestibility [17]. Both tannins [18,19] and saponins [11,20] have also been shown to decelerate ruminal protein degradation and, therefore, prevent the formation of (excessive) ammonia, although their mode of action differs. Tannins bind to proteins at a ruminal pH, thus preventing access by microbes. Saponins hamper the activity of microbes at different steps of protein degradation. Though both tannins and saponins, when tested separately, have been reported to be effective against ruminal methane and ammonia emissions, their efficiency in combination (which could be additive, synergistic, or antagonistic) has rarely been investigated. In a previous study [21], the effects of combining tannin and saponin extracts on in vitro rumen fermentation were tested, but this was performed exclusively with similar proportions between the extracts, and methane formation was not measured. To the best of our knowledge, there has been no study that has attempted to combine plant extracts rich in tannins and saponins in different proportions and to test their effects on rumen methanogenesis.

The objective of the present research was therefore to investigate the effects of combining plant extracts rich in tannins and saponins at varying proportions on in vitro methanogenesis and rumen fermentation. The following hypotheses were tested: (1) Extracts from *Swietenia mahogani* leaves rich in tannins and from *Sapindus rarak* fruits rich in saponins are effective at decreasing ruminal methane and ammonia formation. (2) When provided in combination, tannins and saponins will act synergistically in this respect owing to the different principles of action. These hypotheses were tested in vitro at varying proportions of tannins and saponins, as well as in two contrasting diet types—a high-forage diet and a high-concentrate diet. The two sources of tannins and saponin were chosen because the two plants are particularly rich in these constituents [22,23]. The extraction of these plant sources at various solvent combinations has not been previously performed.

## 2. Materials and Methods

### 2.1. Collection of Plants and Their Enrichment in Tannins and Saponins by Extraction

*Swietenia mahogani* leaves and *Sapindus rarak* fruits were obtained from the area of the Indonesian Research Center for Animal Production, Ciawi Bogor, Indonesia. These plant materials are commonly used as traditional human medicine and for washing “batik,” a traditional Indonesian cloth, respectively. Shortly after collection, the plant materials were oven-dried at 50 °C for 24 h and subsequently ground to pass through a 0.5-mm sieve. The materials were subjected to various types of solvent extraction to identify the method that resulted in the most concentrated tannin and saponin extracts. The solvents used were water, acetone, and methanol, either alone or in 3:1, 1:1, or 1:3 mixtures of either methanol or acetone with water. Ten milliliters of each solvent or solvent combination were inserted into a test tube containing 0.5 g of the ground plant materials. The tube was then placed in an ultrasonic water bath (Barnstead/Lab Line Aqua Wave 9377, E60H, Germany), and the solvent was allowed to extract tannins or saponins for 20 min at room temperature. Each sample was then centrifuged (Thermo Scientific IEC Centra CL2 Centrifuge, Fisher Scientific Pte Ltd., Singapore) at 3000 *g* and 4 °C for 10 min. This procedure was repeated twice, and the supernatants were combined and subsequently measured for tannin and saponin concentrations [24,25]. All types of extractions were conducted in three replicates. The solvent types that resulted in the highest tannin and saponin concentrations were subsequently used to prepare the extracts for the in vitro rumen fermentation experiment. For that purpose, the organic solvent was removed by a rotary evaporator (Buchi Rotavapor R-200, Germany), followed by freeze-drying for 24 h to obtain dry extracts. The dried extracts were then solubilized in distilled water and subjected to in vitro rumen incubation, together with substrates and a rumen‒buffer solution.

### 2.2. In Vitro Rumen Fermentation

The two basal diets used as substrates in the in vitro test differed in their forage-to-concentrate proportions. One was a high-forage (HF) diet (forage-to-concentrate ratio: 7:3); the other was a high-concentrate (HC) diet (forage-to-concentrate ratio: 3:7). The forage used was Napier grass (*Pennisetum purpureum*), which was freshly collected, oven-dried at 50 °C for 24 h, and then ground to pass through a 1-mm sieve size for further nutrient analysis and in vitro incubation. This screen size was used by the in vitro method employed in the present experiment [26]. Furthermore, a 1-mm screen size has been recommended for in vitro batch culture experiments that assess enteric methane mitigation in ruminants [27]. The concentrate was purchased as a dairy cow concentrate from a local commercial supplier (CV Tani Mulya, Bogor, Indonesia). It was composed of rice bran, cassava pomace, palm kernel cake, copra meal, coffee husk, molasses, NaCl, and a vitamin and mineral mix. The analyzed nutrient composition results of the ingredients are given in Table 1, and the calculated composition of the complete HF and HC diets is also presented. 

The in vitro rumen fermentation was simulated according to the procedure of Theodorou et al. [26]. One gram of substrate was transferred into a 175-mL serum bottle together with 100 mL of buffered rumen fluid as the incubation medium. The experimental treatments consisted of 10 mg/mL of two different substrates (either the HF or HC diets) and five plant extracts rich in tannin and saponin treatments, with the extracts added at 2 mg/mL. In addition, the two basal diet treatments were incubated without extracts, resulting in 12 treatments in total. The five extract treatments comprised 100% plant extracts rich in tannins (T_100_) or saponins (S_100_) and three combinations of each with 3:1 (T_75_S_25_), 1:1 (T_50_S_50_), or 1:3 (T_25_S_75_) of T and S, respectively. These extracts were crude extracts (so may have contained other components), not in the purified forms of tannins or saponins. Following the work of Menke et al. [28], the incubation medium was composed of a bicarbonate buffer solution, a macro-mineral solution, a micro-mineral solution, resazurin, distilled water, a reducing solution, and rumen fluid. The ratio between rumen fluid and the incubation medium was 1:4 (v/v). Rumen fluid, together with rumen solid particles, was collected just before the morning feeding from a rumen-cannulated Holstein Friesian cow cared for following the guidelines of the Federation of Animal Science Societies [29] and housed at the Indonesian Research Center for Animal Production, Ciawi, Bogor. The cow was fed with Napier grass and concentrate (7:3 w/w), similar to the substrates used in the in vitro experiment, throughout the whole experimental period in order to minimize the variation among different in vitro incubation runs. Rumen fluid and solid particles were immediately transported into the laboratory (less than 15 min after collection) and subsequently filtered through four layers of muslin before use. During preparation, the buffered rumen fluid was continuously flushed with CO_2_ to maintain its anaerobic environment. Serum bottles were sealed with butyl rubber stoppers and aluminum crimp seals shortly before starting the incubation. Incubation was carried out in a water bath maintained at 39 °C for 48 h. Gas production was vented and recorded at 1, 2, 3, 4, 6, 8, 12, 16, 24, 30, 36, and 48 h after incubation, and the bottles were manually shaken after each gas production reading. The in vitro incubation was performed in four runs, each in a different week. Each treatment per incubation run was represented by two serum bottles. Three bottles per run without any substrate but containing buffered rumen fluid were also incubated to serve as blanks.

### 2.3. Chemical Composition, Fermentation Product and Microbial Analyses

Total phenols and total tannins were measured in the *S. mahogani* extracts obtained according the procedure used by Makkar [24] by employing the Folin‒Ciocalteu method. Polyvinyl polypyrrolidone (PVPP) was used to separate tannin phenols from non-tannin phenols. The absorbance was measured using a spectrophotometer (UV–Vis, U-1800, 5930482, High Technology Corporation, Tokyo, Japan) at a wavelength of 724 nm. Tannic acid was used as the standard for quantification of total extractable phenols and total tannins. The analysis of total saponins in the *S. rarak* extracts was performed according to the work of Hiai and Nakajima [25] and calibrated against a diosgenin standard (Sigma-Aldrich D1634, Sigma Aldrich Chemie GmbH, Steinheim, Germany). Briefly, the sample was added to 0.2 mL of vanillin, 0.25 mL of ethanol, and 2.5 mL of 72% H_2_SO_4_, and then it was vortexed. Afterwards, it was heated in a water bath (Watson Victor Ltd., Bw6t, Watson Victor Limited, New Zealand) at 60 °C for 10 min. After cooling, the absorbance was determined using the same spectrophotometer as applied for tannins, but at a wavelength of 544 nm. 

The substrates were analyzed for dry matter (DM), total ash, crude protein [30], neutral detergent fiber (NDF), acid detergent fiber (ADF), and acid detergent lignin [31]. No α-amylase was used for the NDF analysis, and NDF and ADF were expressed without residual ash. Fermentation gas production was recorded by using calibrated glass syringes. Methane concentration was measured in the intervals in the gas release by using the CO_2_ trapping method with NaOH according to a study by Fievez et al. [32]. After 48 h of incubation, the fermentation residue was filtered and dried in an oven at 105 °C for 24 h. In addition to DM, total ash was determined according to Association of Official Analytical Chemists (AOAC) [30]. Amounts of dry matter and organic matter supply and residue were used to calculate the in vitro dry matter and organic matter degradability (IVDMD and IVOMD, respectively). The ammonia concentration was measured in the incubation liquid obtained after 48 h by the Conway micro-diffusion technique, as described by Nocek et al. [33]. The volatile fatty acid (VFA) profile was obtained by injecting the incubation liquid into a gas chromatograph (GC 8A, Shimadzu Corp., Kyoto, Japan) following the procedure of Jayanegara et al. [16]. The incubation liquid was fixed with a Hayem solution (2.5 mg/mL of HgCl_2_, 25 mg/mL of Na2_S_O_4_, and 5.0 mg/mL of NaCl) prior to counting the bacteria. To count the protozoa, the incubation liquid was treated with 1:10 diluted formaldehyde (400/100 *w/v* in water). Bacteria and protozoa populations were counted by using Bürker counting chambers (Blau Brand, Wertheim, Germany) with 0.02 and 0.1 mm depths, respectively.

### 2.4. Statistical Analysis and Calculations

Data were subjected to an analysis of variance test. The test of the extraction procedures for tannins and saponins was based on a completely randomized design with three replicates per treatment. The in vitro rumen fermentation experiment was based on a randomized complete block design with four replicates (runs) per treatment. Each replicate (run) was represented by the average of two incubation bottles. Different batches of rumen fluid (runs) served as the block. The randomized complete block design was chosen due to insufficient space in the water bath for incubating all the serum bottles at once. When there was a significant effect at *p* < 0.05 of any of the treatments, Duncan’s multiple range test was conducted for multiple comparisons among treatment means. Bacteria and protozoa counts were transformed into their logarithmic values prior to the analysis of variance. Associative effects between plant extracts rich in tannins and saponins were calculated as the difference between observed values (obtained by the measurements) and expected values (arithmetic means of the values obtained with incubations with tannin or saponin extracts, exclusively). These results are presented as percentage of the expected values [23]. A paired *t*-test was conducted to identify significance between the observed and the expected values. All statistical analyses were performed by using the IBM SPSS statistical software version 20.0. The result tables give the standard errors of the mean (SEM) and *p*-values.

## 3. Results and Discussion

### 3.1. Effect of Type of Solvent on the Efficiency of Extraction of Tannins and Saponins

**Figure 1:**
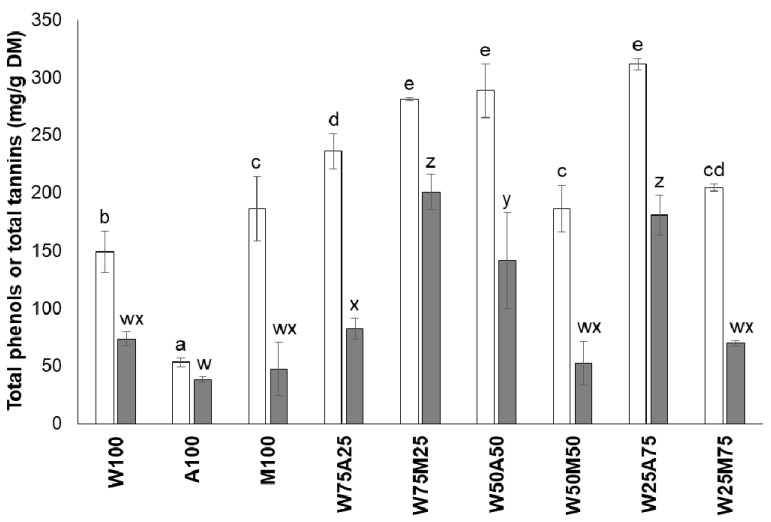
Concentrations of phenols (empty bars) and tannins (grey bars) in the DM of *Swietenia mahogani* leaf extracts obtained with different solvents (W: water; M: methanol; A: acetone; and numbers describe percentages of the respective solvents used). Bars carrying no common letter within the same parameter are different at *p* < 0.05.

The extraction of phenols and tannins by either water (W), acetone (A), or methanol (M) alone revealed a low recovery, although there were differences among the three solvents (Figure 1). The order of extraction efficiency for phenols was M > W > A, whereas it was W > M > A for tannins. Thus, it seems that solvents with a higher polarity have a better ability to extract phenols and tannins from plant matrices than solvents with a lower polarity. Confirming our findings, Iqbal et al. [34] found that the use of a solvent with a high polarity, such as methanol, increased the efficiency of the extraction of phenols from *Artemisia annua* leaves. Furthermore, these authors found that the order of different solvents with regard to their ability to extract phenols was M > W > ethanol > A > chloroform > hexane.

Combining W with either A or M improved the extraction efficiency for phenols and tannins (*p* < 0.05), with a few exceptions. The extraction of phenols was particularly efficient (i.e., >250 mg/g) with W_75_M_25_, W_50_A_50_, and W_25_A_75_, and the extraction with W_75_M_25_ and W_25_A_75_ resulted in a tannin concentration of >150 mg/g DM. An explanation for the higher extraction efficiency of the solvent mixtures compared to the single solvents lies in the chemical structure of the tannins. They contain both hydrophilic (polar) and hydrophobic (nonpolar) groups in their structures, i.e., hydroxyl groups and aromatic phenolic groups, respectively [35]. Makkar [24], for instance, suggested W_30_A_70_ for extracting tannins from various plant sources. Though this could be seen as a starting point for extracting tannins, efficiency studies for individual tannin sources would better account for the diversity of the chemical structures of tannins occurring across different plants [35]. In the present study, W_75_M_25_ and W_25_A_75_ were the most effective solvent mixtures. W_75_M_25_ was eventually preferred for extracting tannins from *S. mahogani* because it required much less organic solvent than the other mixture.

**Figure 2:**
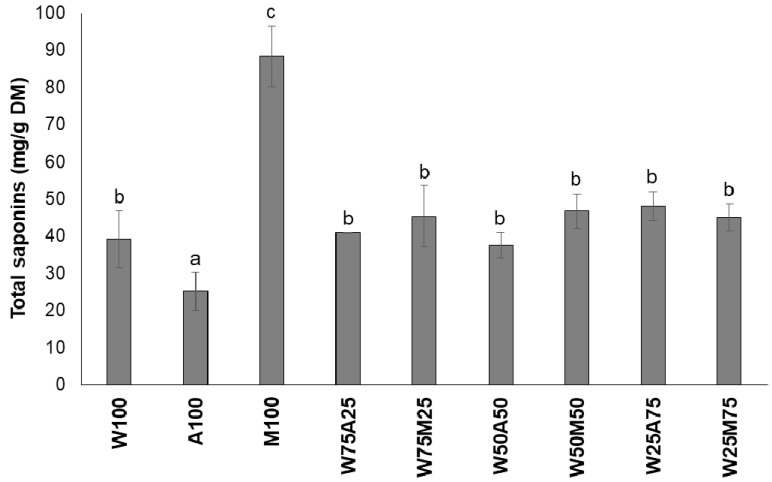
Saponin concentrations in the DM of *Sapindus rarak* fruit extracts obtained with different solvents. Bars carrying no common letter are different at *p* < 0.05.

The efficiency of the extraction of saponins from the *S. rarak* fruits was best achieved by using methanol as the exclusive solvent (M_100_). The saponin concentration then was higher (*p* < 0.05) than with any of the other solvents tested (Figure 2). Saponins contain polar glycones and nonpolar aglycones (sapogenin) [15]. Though this chemical diversity is similar to that of the tannins, the extraction efficiency of saponins from *S. rarak* did not increase when using mixtures of solvents. The recommendation by Makkar et al. [36] of using W_50_M_50_ to extract saponins from plant samples was not applicable to *S. rarak* fruits. This was in agreement with a study conducted by Wina et al. [22], where M_100_ was also used to extract saponins from *S. rarak* fruits. Therefore, M_100_ was used as the solvent to extract saponins from the *S. rarak* fruits for the in vitro experiment.

### 3.2. Effects of Basal Diet Type

Total gas production over 24 and 48 h was higher when incubating the HC diet compared to the HF diet (*p* < 0.05; Table 2), as expected from the lower contents of NDF and ADF (Table 1) and, thus, likely higher contents of non-fiber carbohydrates, especially starch. Consistent with this, Anele et al. [37] observed that high-starch, low-fiber barley grain caused a higher in vitro gas production than low-starch, high-fiber barley grain. Furthermore, the proportion of soluble DM, the fraction of DM insoluble but degradable in the rumen, and the DM disappearance rate were consistently higher in the high-starch compared to the low-starch barley in that study. Consistent with the higher total gas production, IVDMD and IVOMD were higher (*p* < 0.001) with the HC diet than the HF diet (Table 3), whereas bacteria and protozoa counts were not affected by the basal diet type. Bacteria population were considered to be low in this study since they were less than 10^9^ cells/mL. This condition, however, apparently did not lead to a substantial problem, as can be seen from the normal data of rumen fermentation and degradability parameters. Consistent with the higher dietary crude protein content, the HC diet resulted in a higher incubation liquid ammonia concentration (*p* < 0.001; Table 3), which was in line with the lower fiber content in a lower methane-to-total gas ratio (*p* < 0.01; Table 2). It is well-known that increasing the concentrate proportion in an HC diet will lower its methane emissions [38,39,40]. In that case, less total fiber and less digestible fiber is available, which leads to smaller amounts of hydrogen for methanogenesis [40].

### 3.3. Effects of Plant Extracts Rich in Tannins and Saponins and Their Interaction with Diet Type

The addition of plant extracts rich in tannins alone (T_100_) did not decrease the total gas production when incubating both the HF and HC diets in comparison to the control (Table 2). The exclusive addition of the plant extracts rich in saponins (S_100_) reduced (*p* < 0.05) the total gas production from the HF diet within 24 h of incubation (*p* < 0.05), but the effect disappeared within 48 h of fermentation. No significant effect of S_100_ was observed when added to the HC diet. 

For a comparison of the effects of tannins and saponins against methane emission, the proportion of total gas was chosen. A decrease in absolute methane emissions with a concomitant decline in total gas production would likely only reflect the effect of an impaired nutrient degradation and not be based on a real antimethanogenic effect. The addition of plant extracts rich in tannins or saponins individually decreased (*p* < 0.05) the methane proportion of total gas in both the HF and HC diets (Table 2). This was consistently observed after 24 and 48 h of incubation. There was an interaction between diet and extract addition (*p* < 0.001) for the methane proportion of the total gas. This was due to the lower magnitude of the methane decrease after the addition of extracts to the HC diet (8.4% and 4.6% after 24 h incubation with plant extracts rich in tannins and saponins, respectively) compared to the HF diet (20.2% and 16.2%, respectively). Different from oils [41], plant secondary compounds are obviously more efficient in forage-based diets. In the present study, the decrease in relative methane formation caused by the addition of the tannin extract was more pronounced (*p* < 0.001) than that found with the saponin extract. However, it was also associated with a decline in the degradability of substrate in the rumen fluid, as was obvious from the lower IVDMD and IVOMD as compared to the control (Table 3). Tannins are able to form complexes with proteins and carbohydrates (both fiber and non-fiber types) through hydrogen or hydrophobic bonds or both, thus making the components less available to microbial degradation and fermentation [42]. Such a condition leads to a lower hydrogen formation, which is relevant because hydrogen is a main substrate for methanogenesis [1]. Tannins may also decrease methane emissions by lowering the methanogen population. In a previous study [16], it was demonstrated that the addition of purified hydrolysable (from chestnut and sumac) and condensed tannins (from mimosa and quebracho) at 1 mg/mL of the incubation medium reduced the methanogen population by 22.3–36.7% in comparison to the control. The methanogen count was not measured in the present experiment.

The reduction in relative methane emissions due to the addition of the saponin extract was associated with a lower protozoa population in comparison to the control in both the HC and HF diets (*p* < 0.05) (Table 3). Saponins are known to possess antiprotozoal effects through their interaction with cholesterol in the protozoal cell membrane, which leads to cell lysis; this is true for both triterpenoid and steroid saponins [15]. Since some methanogens live symbiotically with protozoa and protozoa provide hydrogen as a substrate for methane formation [5], any reduction in the protozoa population may decrease the methanogen population and methanogenesis. A lower methanogen population due to the addition of saponins has also been observed in other studies [43,44]. Another plausible mechanism by which saponins decrease methane emissions is by lowering the hydrogen supply from bacteria and fungi [45]. The addition of the saponin extract simultaneously decreased the proportion of acetate and increased the proportion of propionate compared to the control (*p* < 0.05; Table 4). Such shifts in VFA profiles lead to further reductions in methane emissions since, stoichiometrically, the formation of acetate from monosaccharide fermentation produces hydrogen, whereas the formation of propionate requires hydrogen [1].

The addition of exclusively plant extracts rich in tannins or saponins reduced the ammonia concentration in the incubation medium of both the HF and HC diets (*p* < 0.05; Table 3). The decline in the ammonia concentration was more pronounced in the high-concentrate diet than in the high-forage diet (interaction, *p* < 0.001), being 26.7% and 17.9% with plant extracts rich in tannins and saponins, respectively, compared to 17.2% with the HF diet and any of the extracts. Plant extracts rich in tannins and saponins were thus able to effectively decrease the ruminal ammonia concentration. The greater potential, especially for tannins, for reducing the ammonia concentration when incubating the HC vs. the HF diets was likely due to the greater dietary crude protein content, as a lower proportion was utilized by the microbes to form their own protein. Furthermore, more protein was available to be rendered inaccessible and degraded to ammonia by the microbes due to binding by the tannins.

The lower IVDMD and IVOMD values found in both diets in response to the addition of plant extracts rich in tannins and saponins may be associated with decreasing counts of protozoa, and adverse effects of tannins and saponins are known [14,15]. A decline was not seen for the bacterial count. One reason for this could be the peptidoglycan layer, which is present in the cell wall of bacteria but not of protozoa and which could make bacteria more resistant to plant secondary compounds.

It has to be mentioned that fermentation products and microbial counts were analyzed only after 48 h of incubation—not after 24 h, as for gas production. However, even though gas production values were not twice as high after 48 h as those after 24 h, the treatment differences were similar, suggesting that these effects were likely similar after 48 h in all other traits as well.

### 3.4. Effects of Combining Plant Extracts Rich in Tannins and Saponins

Combinations of plant extracts rich in tannins and saponins generally acted additively on the total gas production, methane concentration, IVDMD, IVOMD, and total VFA. Only few associative effects between the extracts, i.e., effects where combinations deviated from the average of those obtained with the extract alone, were observed (Table 5). Concerning methane proportion of total gas, the T_25_S_75_ combination was more effective (*p* < 0.05) than expected from incubating plant extracts rich in tannins or saponins alone. With regard to ammonia concentration, there were much clearer associative effects of the combined addition of plant extracts rich in tannins and saponins—with both the HF and HC diets. The T_25_S_75_, T_50_S_50_, and T_75_S_25_ extracts resulted in a lower ammonia concentration (*p* < 0.05) than that expected from the average of plant extracts rich in tannins and saponins incubated alone. One exception was the T_25_S_75_ extract added to the HC diet. Tannins and saponins have different mechanisms by which they decrease ruminal ammonia formation, and they therefore seem to act more intensively when given in combination. By binding protein molecules in feed, tannins slow down the rate and extent of protein degradation and amino acid deamination to ammonia [23]. By contrast, saponins lower ammonia concentration, especially via direct antimicrobial effects; these include antiprotozoal effects [15]. Protozoa intensively predate bacteria, thus reducing the efficiency of total microbial protein synthesis and, thereby, the removal of ammonia. On the other hand, saponins inhibit proteolytic rumen bacteria such as *Streptococcus bovis*, *Butyrivibrio fibrisolvens*, and *Prevotella bryantii* [46,47].

## 4. Conclusions

Tannin and saponin additions in the form of extracts of *Swietenia mahogani* and *Sapindus rarak* were shown to be effective in the mitigation of ruminal methane and ammonia formation in every basal diet type—though they were more effective in the forage-based diet in the case of methane and in the concentrate-based diet in the case of ammonia. A particularly interesting finding was that the presence of the frequent additivity of the effects of tannins and saponins which, when combined, made them predictable from the effects of the pure extracts. However, both additives obviously interacted synergistically in protecting part of the protein from ruminal degradation, as could be seen from the particularly low rumen ammonia concentration with combinations. Combinations would therefore need lower amounts of such costly extracts in abating N emissions through concomitantly losing less N as volatile urine. A drawback is that both extracts impeded feed degradability. How far this translates into lower performance and affects the extent to which methane and N emissions can be recovered with combinations of the two plant secondary compounds in vivo remains to be investigated. 

## Figures and Tables

**Table 1:** Nutrient composition of dried Napier grass, concentrate, and high-forage (HF) and high-concentrate (HC) diets (in mg/g dry matter (DM)).

Item	Napier Grass	Concentrate	HF Diet	HC Diet
Organic matter	881	942	899	924
Crude protein	90	184	118	156
Neutral detergent fiber	656	270	540	386
Acid detergent fiber	447	117	348	216
Acid detergent lignin	94	50	81	63

HF: 700 mg/g Napier grass and 300 mg/g concentrate; and HC: 300 mg/g Napier grass and 700 mg/g concentrate.

**Table 2:** In vitro gas production and methane proportion of total fermentation gas, as obtained with fermenting either the HF or the HC diets supplemented with varying proportions of plant extracts rich in tannins (T) and saponins (S).

Diet	Extract	Total Gas (mL/g DM)		Methane (mL/L Total Gas)	
		24 h	48 h	24 h	48 h
HF	C	186 ^b^	233 ^a^	277 ^d^	286 ^d^
	T_100_	182 ^a,b^	242 ^b^	221 ^a,b^	239 ^b,c^
	S_100_	173 ^a^	233 ^a^	232 ^b,c^	233 ^a,b^
	T_25_S_75_	179 ^a,b^	245 ^b^	218 ^a^	226 ^a^
	T_50_S_50_	184 ^b^	247 ^b^	221 ^a,b^	229 ^a,b^
	T_75_S_25_	187 ^b^	243 ^b^	221 ^a,b^	229 ^a,b^
HC	C	236 ^d^	275 ^c^	238 ^c^	247 ^c^
	T_100_	232 ^d^	281 ^c,d^	218 ^a^	229 ^a,b^
	S_100_	229 ^c,d^	281 ^c,d^	227 ^a,b,c^	236 ^a,b^
	T_25_S_75_	221 ^c^	277 ^c,d^	223 ^a,b^	230 ^a,b^
	T_50_S_50_	235 ^d^	287 ^e^	225 ^a,b^	232 ^a,b^
	T_75_S_25_	236 ^d^	285 ^d,e^	223 ^a,b^	232 ^a,b^
SEM		2.8	2.4	2.1	2.0
*p*-value					
Diet		<0.001	<0.001	0.003	0.002
Extract		0.001	<0.001	<0.001	<0.001
Diet × extract		0.442	0.182	<0.001	<0.001

Means carrying no common superscript within the same column are different at *p* < 0.05. HF: 700 mg/g Napier grass and 300 mg/g concentrate; HC: 300 mg/g Napier grass and 700 mg/g concentrate; and C: unsupplemented control. Indices describe percentages of the respective extracts.

**Table 3:** In vitro dry matter and organic matter degradability (IVDMD and IVOMD, respectively), ammonia concentration, log bacteria, and log protozoa counts measured after 48 h of incubation with the HF or HC diets, supplemented with varying proportions of plant extracts rich in T and S.

Diet	Extract	IVDMD	IVOMD	Ammonia	Bacteria	Protozoa
		(mg/g)	(mg/g)	(mmol/L)	(log/mL)	(log/mL)
HF	C	629 ^d^	704 ^f^	24.4 ^f^	8.56	6.04 ^e^
	T_100_	463 ^a^	449 ^a,b^	20.2 ^d^	8.55	6.03 ^d,e^
	S_100_	520 ^b^	505 ^c^	20.2 ^d^	8.48	5.24 ^a^
	T_25_S_75_	47 9 ^a^	483 ^b,c^	18.7 ^c^	8.53	5.68 ^b^
	T_50_S_50_	470 ^a^	444 ^a^	16.6 ^a^	8.47	5.62 ^b^
	T_75_S_25_	467 ^a^	477 ^a,b,c^	16.5 ^a^	8.38	5.94 ^c,d^
HC	C	674 ^e^	695 ^f^	26.2 ^g^	8.23	5.98 ^c,d,e^
	T_100_	526 ^b^	548 ^d^	19.2 ^c^	8.54	6.01 ^c,d,e^
	S_100_	604 ^c,d^	609 ^e^	21.5 ^e^	8.65	5.26 ^a^
	T_25_S_75_	598 ^c^	608 ^e^	20.7 ^d^	8.41	5.61 ^b^
	T_50_S_50_	549 ^b^	569 ^d^	18.8 ^c^	8.53	5.68 ^b^
	T_75_S_25_	550 ^b^	564 ^d^	17.6 ^b^	8.58	5.92 ^c^
SEM		8.4	13.2	0.32	0.034	0.041
*p*-value						
Diet		<0.001	<0.001	<0.001	0.964	0.394
Extract		<0.001	<0.001	<0.001	0.790	<0.001
Diet × extract		0.033	<0.001	<0.001	0.277	0.223

Means carrying no common superscript within the same column are different at *p* < 0.05. HF: 700 mg/g Napier grass and 300 mg/g concentrate; HC: 300 mg/g Napier grass and 700 mg/g concentrate; and C: unsupplemented control. Indices describe percentages of the respective extracts.

**Table 4:** Volatile fatty acid (VFA) profiles measured after 48 h of incubation of the HF or HC diets supplemented with varying proportions of plant extracts rich in T and S.

Diet	Extract	Total VFA	C_2_	C_3_	C_4_	*iso*C_4_	C_5_	*iso*C_5_	C_2_/C_3_
		(mmol/L)	(%)	(%)	(%)	(%)	(%)	(%)	
HF	C	84.6 ^b,c^	54.9 ^b,c^	26.2 ^a^	9.80 ^d,e^	4.40 ^c^	1.54 ^d,e^	3.20 ^c^	2.10 ^d,e^
	T_100_	80.8 ^a,b^	55.7 ^b,c^	27.9 ^a,b^	8.82 ^a,b,c^	4.15 ^b,c^	1.30 ^b,c,d,e^	2.12 ^a^	2.00 ^c,d,e^
	S_100_	77.7 ^a,b^	52.6 ^a,b^	32.5 ^e^	8.15 ^a^	3.36 ^a^	1.06 ^a,b,c^	2.05 ^a^	1.63 ^a^
	T_25_S_75_	83.4 ^a,b,c^	55.2 ^b,c^	30.3 ^c,d^	8.10 ^a^	3.49 ^a,b^	0.96 ^a,b^	1.92 ^a^	1.84 ^a,b,c^
	T_50_S_50_	91.4 ^b,c^	56.8 ^c^	28.9 ^b,c^	8.08 ^a^	3.50 ^a,b^	0.85 ^a^	1.85 ^a^	1.97 ^c,d,e^
	T_75_S_25_	63.1 ^a^	56.0 ^c^	29.8 ^b,c,d^	8.32 ^a,b^	3.76 ^a,b,c^	0.96 ^a,b^	2.08 ^a^	1.90 ^c,d^
HC	C	79.9 ^a,b^	55.4 ^b,c^	26.0 ^a^	10.3 ^e^	3.83 ^a,b,c^	1.45 ^c,d,e^	3.00 ^b,c^	2.14 ^e^
	T_100_	76.0 ^a,b^	55.4 ^b,c^	27.8 ^a,b^	9.26 ^c,d^	3.76 ^a,b,c^	1.14 ^a,b,c,d^	2.38 ^a,b^	2.00 ^c,d,e^
	S_100_	102 ^c^	51.4 ^a^	31.4 ^d,e^	8.52 ^a,b,c^	3.53 ^a,b^	1.69 ^e^	2.33 ^a^	1.66 ^a,b^
	T_25_S_75_	84.8 ^b,c^	53.8 ^a,b,c^	29.7 ^b,c,d^	9.08 ^b,c,d^	3.67 ^a,b^	1.28 ^b,c,d,e^	2.46 ^a,b^	1.82 ^a,b,c^
	T_50_S_50_	90.9 ^b,c^	52.6 ^a,b^	29.2 ^b,c^	9.31 ^c,d^	3.89 ^a,b,c^	1.32 ^b,c,d,e^	2.51 ^a,b^	1.88 ^b,c,d^
	T_75_S_25_	86.7 ^b,c^	53.6 ^a,b,c^	28.8 ^b,c^	9.17 ^c,d^	3.89 ^a,b,c^	1.17 ^a,b,c,d^	2.40 ^a,b^	1.78 ^a,b,c^
SEM		2.41	0.380	0.402	0.139	0.075	0.048	0.076	0.036
*p*-value									
Diet		0.133	0.016	0.334	<0.001	0.999	0.003	0.013	0.429
Extract		0.356	0.022	<0.001	<0.001	0.029	0.007	<0.001	<0.001
Diet × extract		0.046	0.258	0.917	0.491	0.185	0.031	0.385	0.724

Means carrying no common superscript within the same column are different at *p* < 0.05. HF: 700 mg/g Napier grass and 300 mg/g concentrate; HC: 300 mg/g Napier grass and 700 mg/g concentrate; C: unsupplemented control; and VFA: volatile fatty acids. Indices describe percentages of the respective extracts.

**Table 5:** Associative effects ^†^ of the combinations between plant extracts rich in T and S added to the HF or HC diets.

Diet	Extract	Gas 24 h (mL/g DM)	Gas 48 h (mL/g DM)	CH_4_ 24 h (mL/L gas)	CH_4_ 48 h (mL/L gas)	IVDMD 48 h (mg/g)	IVOMD 48 h (mg/g)	Total VFA 48 h (mmol/L)	Ammonia 48 h (mmol/L)
HF	T_25_S_75_	4.4	4.1	−4.9 **	−3.2 *	−5.6	−1.7	7.8	−8.2 ***
	T_50_S_50_	3.9	3.0	−2.0	−1.9	−4.6	−6.9	−21.1 *	−16.3 ***
	T_75_S_25_	3.7 *	0.9	1.3	0.1	−2.2	3.0	1.3	−19.6 ***
HC	T_25_S_75_	−4.6	−2.1	−0.6	−1.8	2.2	2.3	8.1	−1.5
	T_50_S_50_	2.4	3.0 **	1.0	−0.1	−3.0	−1.8	12.2	−6.8 **
	T_75_S_25_	2.1	2.0	1.3	0.4	0.9	0.2	11.7	−13.5 **

* *p* < 0.05; ** *p* < 0.01; *** *p* < 0.001; ^†^ Difference between observed values (obtained by measurements) and expected values (arithmetic means of the values obtained with incubations with exclusively plant extracts rich in tannins or saponins) in percent of the expected values; HF: 700 mg/g Napier grass and 300 mg/g concentrate; HC: 300 mg/g Napier grass and 700 mg/g concentrate; CH_4_: methane; DM: dry matter; IVDMD: in vitro dry matter degradability; IVOMD: in vitro organic matter degradability; and VFA: volatile fatty acids. Indices describe percentages of the respective extracts.
